# Toxicity of Four Commercial Fungicides, Alone and in Combination, on the Earthworm *Eisenia fetida*: A Field Experiment

**DOI:** 10.3390/toxics13030209

**Published:** 2025-03-14

**Authors:** Tommaso Campani, Ilaria Caliani, Agata Di Noi, Silvia Casini

**Affiliations:** 1 Department of Physical, Earth and Environmental Sciences, University of Siena, Via Mattioli 4, 53100 Siena, Italy; campani@unisi.it (T.C.);; 2 Santa Chiara Lab, University of Siena, Via Valdimontone 1, 53100 Siena, Italy

**Keywords:** agricultural soil, DNA damage, ecotoxicological biomarkers, fungicides, non-target soil organism, sub-chronic exposure

## Abstract

This study investigated the sub-lethal effects of four commercial fungicides—two foliar (Amistar^®^Xtra and Mirador^®^) and two ear fungicides (Prosaro^®^ and Icarus^®^)—applied alone and in combination to wheat crops on caged earthworms (*Eisenia fetida*). We measured biomarkers that included detoxification responses (glutathione S-transferase, GST), oxidative stress levels (lipid peroxidation, LPO, and catalase, CAT), DNA damage (comet assay), energy reserves (lactate dehydrogenase, LDH), and immune response (lysozyme activity, LYS). The absence of significant differences in catalase and lipid peroxidation levels suggested no oxidative stress due to fungicide exposure. However, the foliar fungicide Amistar^®^Xtra induced the highest GST activity and DNA fragmentation, suggesting synergistic effects between its active ingredients and undisclosed co-formulants. Similar effects observed with the Amistar^®^Xtra-Prosaro^®^ mixture confirmed the greater toxicity of Amistar^®^Xtra. This study provides novel insights into the sub-lethal effects of single and combined commercial fungicides on a standard toxicity test organism, shedding light on the ecological implications of fungicide use in agroecosystems and reinforcing the need for pesticide reduction.

## 1. Introduction

Grain and rice farming represent one of the most important agricultural production activities worldwide and are a staple food worldwide [1]. Extensive cultivation and the massive use of plant protection products (PPPs) allowed high wheat yields. These chemical substances are used to control mold diseases, insects, and unwanted weeds, and their management must take into account non-target species and soil communities in general, including earthworms. 

A healthy soil ecosystem is defined by its ability to perform functions essential for ecosystem operation [2]. In this contest, earthworms have an important role because they contribute to its physical, chemical, and biological development and help the agroecosystem maintain its resilience [3]. PPPs, which can interact with the environment through different routes of exposure, such as inhalation and direct skin contact, could cause acute and sublethal toxicological effects such as escape from the contaminated soil or death of the earthworms or negative effects on growth or reproduction rate, with consequent deleterious effects on the whole population [4] and on the productivity of the agroecosystem over a long period [5].

The commercial formulation of agrochemicals combines more than one active ingredient with a solvent and co-adjuvant, often surfactants [6,7]. The toxicity of the PPPs’ active principles has been tested on several organisms, including earthworms, as requested by international protocols of the United Nations within the Globally Harmonized System of Classification and Labeling of Chemicals (GHS) [8]. Sub-lethal and lethal effects of active ingredients on earthworms have been extensively documented under laboratory conditions [9,10,11,12,13,14]. In contrast, limited research has investigated the potential toxicological effects of co-adjuvants on non-target organisms [8,9,10,11,12,13,14,15]. Our previous study demonstrated that co-adjuvants could alter the physicochemical properties of pesticides, thereby influencing their toxicological effects [16]. Also, Nagy et al. [17] reviewed 36 toxicity studies that investigated the potential toxicity of commercial pesticide formulations and their active ingredients (23 on herbicides, 15 on insecticides, and 4 on fungicides) and found that in 24 studies, commercial formulations were more toxic than their active ingredients, which was usually attributed to the presence of co-adjuvants in the formulations. Moreover, knowledge of the ecotoxicological effects of commercial formulations on annelids in field experiments is scarce. While several studies have examined the impact of pollutants, particularly pesticides, on earthworms in agricultural soils [18,19], these investigations primarily focused on behavioral responses such as feeding, avoidance, and locomotion. To our knowledge, no prior research has explored the enzymatic alterations in earthworms exposed in situ experiments to these pesticides. The analyses of the enzymatic alteration at cellular or biochemical levels permit the definition of the impacts of many classes of contaminants at different levels of biological organization. Then, a multi-biomarker approach is a sensitive tool for assessing organisms’ ecotoxicological health status in different taxa [20,21,22,23]. This approach involves enzymatic responses, including xenobiotic metabolization, such as glutathione ***S***-transferase (GST), catalase (CAT), lactate dehydrogenase (LDH), and lysozyme (LYS) activities, lipid peroxidation levels, and DNA alteration. Many PPPs involve GST in the detoxification process, especially fungicides. At the same time, GST works as an antioxidant enzyme, counteracting oxidative stress through the conjugation of GSH with the reactive oxygen species (ROS). Another important enzyme, which regulates the presence of ROS, is catalase (CAT). This enzyme is involved in the transformation of hydrogen peroxide into the cellular environment. The overwhelm of ROS could lead to an accumulation of lipolytic substances, resulting in lipid peroxidation [24]. The determination of lipid peroxidation through the quantification of thiobarbituric acid reactive substance (TBARS) levels is considered a biomarker of oxidative stress widely used in ecotoxicological studies. The final target of the presence of ROS is often DNA; lipolytic substances can disrupt the DNA filament, causing adducts, single- or double-strand breaks, which impairs DNA transcriptions; these DNA alterations can be evaluated by comet assay. A general biomarker that permits the evaluation of cell energy consumption in response to a chemical insult is lactate dehydrogenase (LDH) [25]. The elevated consumption rate of lactate could indicate an alteration in the cell metabolism of the organism. The determination of the lysozyme enzyme allows the evaluation of a possible alteration of the functioning of the immune system since lysozyme is involved in the degradation of the Gram-negative bacterial cell.

The present study aimed to investigate the sub-lethal effects of 4 different commercial fungicides sprayed on wheat crops by the application of a multi-biomarker approach on caged *Eisenia fetida*. This is an epigeic earthworm that inhabits sites with the presence of organic matter [26]. Due to its short reproductive cycle and ease of breeding and management, *Eisenia fetida* is frequently employed as a model organism in standardized toxicity tests and assessments of sublethal effects on soil non-target organisms [27]. To compare the differential sub-lethal toxicities of four commercial fungicides, we used two foliar fungicides, Amistar^®^Xtra and Mirador^®^, and two ear fungicides, Prosaro^®^ and Icarus^®^, alone and in combination. We tested different biomarkers, including detoxification responses (glutathione S-transferase, GST), oxidative stress levels (lipid peroxidation, LPO, and catalase, CAT), DNA alterations (comet assay), energy reserve (LDH), and immunological responses (lysozyme activity).

## 2. Materials and Methods

### 2.1. Plant Protection Products

A field experiment was conducted to evaluate the efficacy of four commercially available fungicides commonly used in cereal crops. Two fungicides were applied during the pseudo stem erection: Amistar^®^Xtra produced by Syngenta, Milan, Italy, with the active ingredients azoxystrobin 200 g/L and cyproconazole 80 g/L and co-formulants (not specified in the label) and Mirador^®^ produced by Adama, Bergamo, Italy, with the active ingredient azoxystrobin 250 g/L and co-formulants (not specified in the label). Two fungicides were applied from the full flag leaf emergence up to mid-flowering: Prosaro^®^, produced by Bayer Crop Science, Latina, Italy with the active principles tebuconazole 125 g/L and prothioconazole 125 g/L and co-formulants (not specified in the label); Icarus^®^, produced by Adama, Bergamo Italy, with the active principle tebuconazole 200 g/L and co-formulants (not specified in the label). The concentration of fungicides applied by bloom spray was established based on the dose reported on the safety label of each PPT for wheat crops (Mirador^®^: 0.9 L/ha; Prosaro^®^: 1 L/ha; Amistar^®^Xtra: 0.8 L/ha; Icarus^®^: 1.25 L/ha).

### 2.2. Plot Description

**Figure 1:**
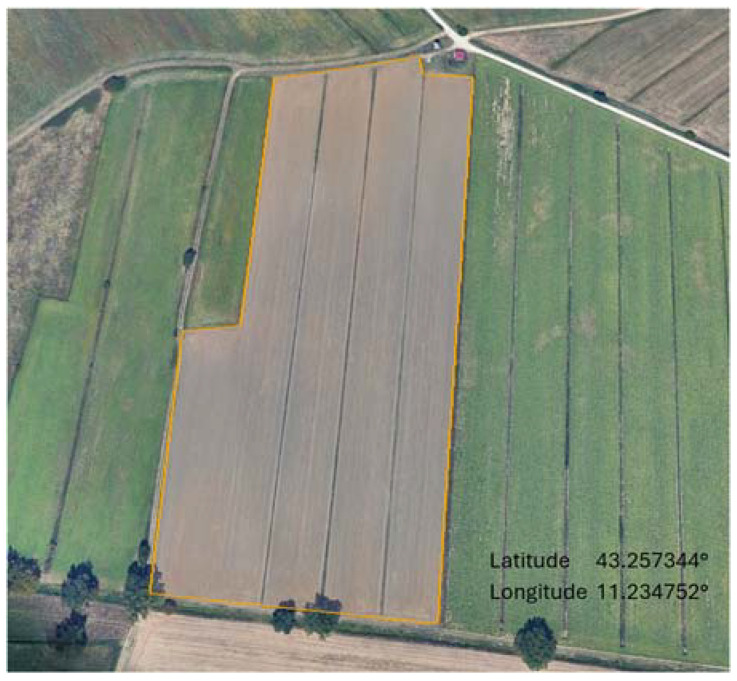
Durum wheat field used (the area inside the yellow line) in the experiment and geographical coordinates.

The experimental field (Figure 1) had a clay loam texture; it exhibited a relatively flat topography with a gentle incline to facilitate drainage and prevent waterlogging. Table 1 describes the grain size and physicochemical analyses of the field.

### 2.3. Experimental Design

**Figure 2:**
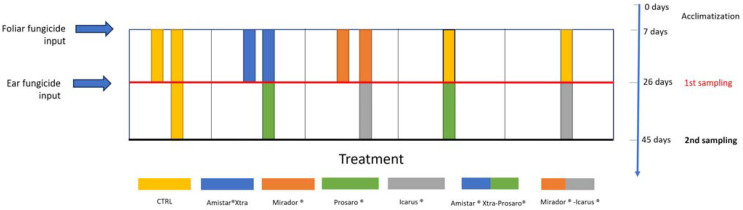
Field experiment layout with plots assigned to different treatments, incorporating sampling time points.

Field experiments were conducted throughout the durum wheat cultivation period (from April to June) by transplanting *E. fetida* specimens in cages before and during treatment with different combinations of the 4 fungicides (Figure 2). In detail, *Eisenia fetida* (Savigny 1826) adults, obtained from a commercial earthworm breeding farm (Lombricoltura Compagnoni, Mandello sul Lario, Como, Italy), were maintained in the laboratory culture at 25 °C for 7 days before the start of the experiments and fed with organic manure. *E. fetida* well-clitelled adults were placed in field cages (a total of 50 animals for each cage, two replicates for each cage) and buried up to the surface of the ground to keep the animals in the soil.

**Figure 3:**
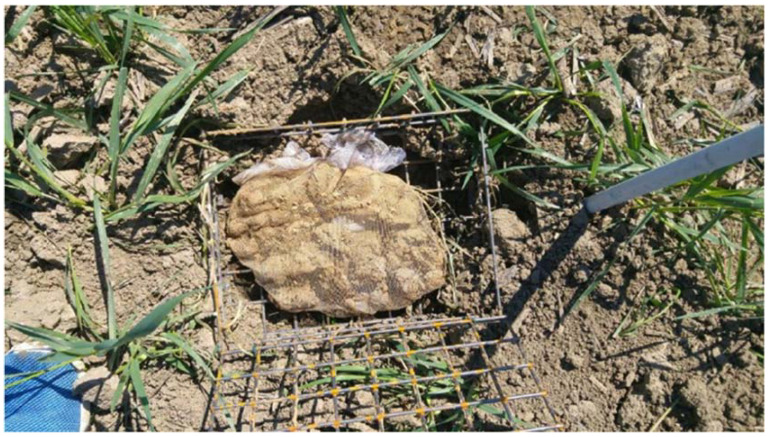
Example of the cage utilized in the experiment. We placed the earthworms in cages kept in mesh bags with moist soil to prevent escape and maintain an optimal environment.

The experiment began after the positioning of the organisms on site to permit their good acclimatization. The experimental field was divided into five plots with a length of approx. 250 m and a width of 7 m each. A buffer zone (approximately 1 m) was provided between the various plots to avoid problems with the drift of contaminants and cross-contamination. Plot 1 served as the control and received no treatment. On the seventh day, all the animals of plots 2 and 4 were, respectively, treated with two foliar fungicides, “Mirador^®^” and “Amistar^®^Xtra. On the nineteenth day, one of the two replicates per plot (25 animals) was removed from the cage (Figure 3) and transferred into the laboratory for analysis, and the other replicates were, respectively, treated with the ear fungicides “Icarus^®^” (M-I: Mirador^®^ + Icarus^®^) and “Prosaro” (A-P: Amistar^®^Xtra + Prosaro^®^). Plots 3 and 5 did not receive treatments until the nineteenth day; then animals from plot 3 were treated with “Prosaro^®^” and plot 5 was exposed to “Icarus^®^”. The organisms were checked daily and kept wet according to weather conditions to ensure their survival. After 19 days, the animals from all the plots were taken to the laboratory and processed. The animals survived entirely to the field exposure, and their viability was checked by visual observation: a scale from zero to three based on individual mobility was used (0 = deceased, 1 = barely perceptible movements, 2 = clear body movements, 3 = no movement deficit) [16].

### 2.4. Biomarkers Analysis

Once in the laboratory, each animal was washed with distilled water and dried with paper. Earthworm coelomocytes were obtained by the method of Eyambe et al. [28]. After the extrusion, the cell suspension was first centrifuged at 150× *g* at 4 °C for 2 min to remove mucus and then for 10 min to recover cells for the comet assay. The animals were then sacrificed by immersion in liquid nitrogen and stored at −80 °C until analysis. The comet assay was evaluated according to the method described by Campani et al. [21]. Fifty cells per sample were examined under an epifluorescence microscope (Olympus BX41, Tokyo, Japan) at 400× magnification. The Komet 5.0 Software (Kinetic Imaging Ltd., London, UK) was used to quantify the DNA fragmentation as a percentage (%) of DNA in the tail. For biochemical analysis, each specimen was homogenized in cold 0.1 M K-phosphate buffer with a Potter homogenizer (0.1 mg/mL). First of all, lipid peroxidation (LPO) was evaluated on a part of the raw homogenate. The rest was centrifuged at 13,200× *g* for 30 min at 4 °C to obtain the post-mitochondrial fraction supernatant (PMS). The PMS was used to determine glutathione *S*-transferase (GST), catalase (CAT), lactate dehydrogenase (LDH), and lysozyme activities. LPO levels were measured spectrophotometrically at 535 nm (AGILENT, Santa Clara, California, United States, Cary UV 60 spectrophotometer) using the procedures by Ohkawa et al. [29] and Bird and Draper [30] and expressed as nmol of thiobarbituric acid reactive substances (ε = 1.56 × 105 M^−1^ cm^−1^) formed. Glutathione *S*-transferase activity was measured in the PMS fraction according to Habig et al. [31], quantifying the conjugation of reduced glutathione (GSH) with 2,4-dinitrochlorobenzene (DNCB) at 342 nm (25 °C) and expressed as nmol DNCB × min^−1^ × mg protein^−1^ (ε = 9.6 × 10^−3^ M cm^−1^). Catalase (CAT) was evaluated in the PMS fraction following the method by Aebi and collaborators [32]. Lactate dehydrogenase (LDH) was evaluated in the PMS fraction according to the method of Menezes et al. [33] and adapted by Caliani et al. [34]. The enzymatic activity was measured by a Multiskan SkyHigh Thermo Scientific microplate reader at 340 nm and expressed as μmol of substrate hydrolysed^−1^ × min. The lysozyme (LYS) activity was measured through the lysis rate of *Micrococcus lysodeikticus* ATCC No. 4698 using the standard turbidity assay described by Keller et al. [35] with a slight modification. The result was expressed in HEL concentration (μg/μL). Protein concentrations were measured spectrophotometrically according to the Bradford method [36].

### 2.5. Statistical Analysis

Data distribution analysis for each biomarker was assessed using the Shapiro–Wilk test. Afterwards, the non-parametric Kruskal–Wallis rank test for population equality was used to assess statistically significant differences between treatment groups. When the Kruskal–Wallis rank test was found to be significant, Dunn’s multiple pairwise comparison tests were performed using the Benjamini–Hochberg adjustment (BH step-up procedure). Spearman’s rank correlation coefficient was also performed. For all tests, a level of significance of 0.05 was assumed. All statistical analyses and graphs were performed using R Studio (version: 2023.06.0), R [37], *Dunn.test* [38], and *ggplot2* [39].

## 3. Results

**Figure 4:**
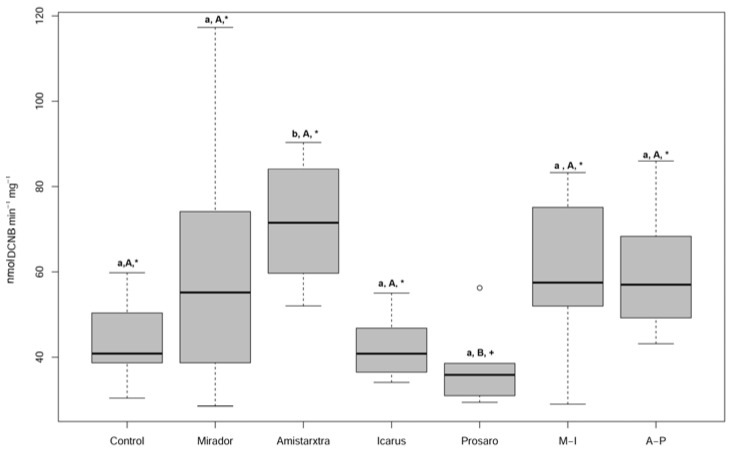
Boxplots of the glutathione *S*-transferase (GST) activity measured in *E. fetida* exposed to the foliar fungicides (Mirador^®^, Amistar^®^Xtra), ear fungicides (Icarus^®^, Prosaro^®^), and binary mixtures (M-I: Mirador^®^-Icarus^®^; A-P: Amistar^®^Xtra-Prosaro^®^). Different lower case letters indicate statistical differences with respect to the control (*p* < 0.05), and different capital letters indicate differences with respect to Amistar^®^Xtra. Different symbols indicate a difference with respect to A-P.

**Figure 5:**
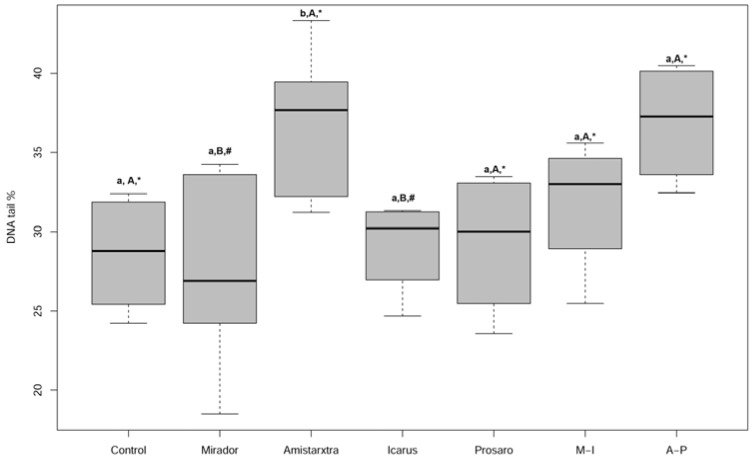
Boxplots of the comet assay, measured in *E. fetida* exposed to the foliar fungicides (Mirador^®^, Amistar^®^Xtra), ear fungicides (Icarus^®^, Prosaro^®^), and binary mixtures (M-I: Mirador^®^-Icarus^®^; A-P: Amistar^®^Xtra-Prosaro^®^). Different lower case letters indicate the statistical differences with respect to the control (*p* < 0.05), and different capital letters indicate differences with respect to Amistar^®^Xtra. Different symbols indicate statistical differences with respect to A-P.

Table S1 reports a summary of all biomarker results (CAT, GST, and LYS activities; LPO, LDH, and comet assay). CAT activity exhibited higher median values in all treatment groups with respect to the control, except for the Icarus^®^ treatment, although these differences were not statistically significant. LPO results indicated minimal levels of peroxidation in cellular membranes, with no statistically significant differences observed among any treatment groups and with respect to the control. GST activity was significantly induced in the Amistar^®^Xtra group compared to the control (Kruskal–Wallis test, χ^2^ = 20.1422, df = 6, *p* < 0.0001, Dunn’s test, *p* = 0.0077) (Figure 4). Additionally, significant differences in GST activity were observed between Amistar^®^Xtra and Prosaro^®^ groups (Dunn’s test, *p* = 0.0080) and between the A-P mix and Prosaro^®^ (Dunn’s test, *p* = 0.0452). Figure 5 illustrates DNA fragmentation in *Eisenia fetida* exposed in the field to four fungicides (Mirador^®^, Amistar^®^Xtra, Icarus^®^, and Prosaro^®^) and two fungicide mixtures (A-P and M-I). Amistar^®^Xtra significantly increased DNA fragmentation compared to Icarus^®^ (Dunn’s test, *p* = 0.039), Mirador^®^ (Dunn’s test, *p* = 0.0332), and the control (Dunn’s test, *p* = 0.0412) as determined using the Kruskal–Wallis test (χ^2^ = 15.7639, df = 6, *p* = 0.02). The A-P mix exhibited a DNA fragmentation similar to that observed with Amistar^®^Xtra, with statistically significant differences compared to Icarus^®^ (Dunn’s test, *p* = 0.0361) and Mirador^®^ (Dunn’s test, *p* = 0.0365). Table S1 shows a reduction in LDH activity following foliar PPP treatments, although these differences were not statistically significant. A statistically significant increase in LDH activity was observed between Icarus^®^ and Mirador^®^ (Kruskal–Wallis test, χ^2^ = 11.4297, df = 5, *p* = 0.04, Dunn’s test, *p* = 0.0268).

## 4. Discussion

When the balance between reactive oxygen species (ROS) and antioxidant activity is lost due to the action of toxic compounds, oxidative stress occurs [40]. This process can impair cell membrane function, damage cell membrane lipids, attack fatty acids, and create lipid radicals, leading to lipid peroxidation of the cell membranes. The results of our study did not observe an increase in lipid peroxidation levels and catalase activity after the exposure to the different PPTs or their mix, indicating that these compounds do not cause oxidative stress in the animals. Differently Ma et al. [12] highlighted the presence of oxidative stress in *Eisenia fetida* after 14 days of exposure to pyraclostrobin, a strobilurin fungicide, observing that CAT activity and MDA levels first increase and later return to the control levels. Wu et al. [41] exposed *E. fetida* to two doses of trifloxystrobin for 7, 28, and 56 days, indicating that this active ingredient can induce oxidative stress (CAT and LPO) and DNA damage. A significant alteration in SOD and CAT activities in earthworms was also observed by Li et al. [42] after exposure to penconazole (a triazole fungicide) for 7 and 14 days. 

Glutathione *S*-transferase is a class of enzymes that catalyze the conjugation of glutathione (GSH) on electrophilic carbon, sulfur, or nitrogen atoms of non-polar xenobiotic substrates, promoting phase II of the detoxification process. Our data about the filter paper test showed no alteration in GST activity in the earthworms exposed to Amistar^®^Xtra, Mirador^®^, and Icarus^®^, while an increase was found after exposure to Prosaro [16]. On the contrary, some authors observed increased GST activity after the earthworms were exposed to soils treated with the active ingredient azoxystrobin [43,44]. The low values observed in our study for Icarus^®^ and Prosaro^®^, containing only triazole-active ingredients, suggest that triazoles alone may not significantly induce GST enzyme activity. This aligns with the findings of our previous study [16]. Induction of GST activity after exposure to Amistar^®^Xtra, which contains the active ingredients azoxystrobin + cyproconazole), was also found. No induction was observed after the exposure to Mirador^®^, which contains only the active ingredient azoxystrobin. These findings suggest an amplification of the sublethal effects of azoxystrobin added to a triazole such as cyproconazole. Moreover, the presence of more active ingredients in the commercial formulation of Amistar^®^Xtra, combined with unknown co-adjuvants, could cause an amplification of the toxicological properties and their effects on non-target species. Compared to the control, the A-P and M-I mixtures exhibited higher GST activity, which was lower than that observed in response to the foliar fungicide Amistar^®^Xtra suggesting a sub-additive activity between the main family of active ingredients, azoxystrobin and triazole. Further studies are needed to better investigate the modes of interaction between active ingredients and co-adjuvants.

DNA is the main cellular component in living organisms that are particularly susceptible to damage from environmental toxic substances [45]. Assessing the DNA strand breaks by the comet assay is a reliable indicator of genotoxicity [46] and has found wide application as a sensitive and rapid visual tool for assessing genotoxic effects in bioindicator organisms [43,44,45,46,47]. Exposure to heavy metals, polycyclic aromatic hydrocarbons [48,49], organochlorines, herbicides [50], and fungicides [20] can cause genotoxic effects in terrestrial organisms. Oxidative stress, caused by ROS in the presence of pesticides, can cause severe damage to cell DNA [51]. Most of the genotoxicity studies on pesticides focus on their active ingredients. In addition, the metabolic processes that various formulants trigger in organisms remain unknown. Some authors link DNA damage to strong oxidative stress caused by exposure to strobilurins [43]. The higher fragmentation values of Amistar^®^Xtra and A-P treatments found in our study could indicate that genotoxic effects are mainly caused by exposure to Amistar^®^Xtra. However, our study does not show evidence of oxidative stress effects but reveals a marked induction of the detoxification system. We could hypothesize that the conjugation process of glutathione with the molecules of azoxystrobin and cyproconazole present in the fungicide Amistar^®^Xtra leads to the activation of metabolites capable of causing DNA damage. Our hypothesis is supported by a statistically significant correlation between GST and comet assay (r = 0.499, *p* = 0.0031). Moreover, the work of Caliani et al. [20] on *Apis mellifera* exposed to field concentrations of Amistar^®^Xtra also displayed the same trend, with induction of the GST activity and increased DNA fragmentation.

Lactate dehydrogenase is an enzyme that catalyzes the reversible conversion of pyruvate to lactate when oxygen is absent or in short supply and NADH recycling and gluconeogenesis. It is a biomarker that assesses the metabolic alterations in organisms exposed to PPPs [52,53]. In general, the results of LDH activity in *E. fetida* can vary depending on the type of pesticide but also on the concentration used; for instance, Rico et al. [54] found in earthworms increased LDH levels up to 213 mg/kg of exposure concentration of tebuconazole but inhibited at the highest one (320 mg/kg). Lammertyn et al. [55] reported no alteration in LDH activity in *E. fetida* after 7 days of laboratory exposure to atrazine (2 mg/kg) and a significant increase after 28 days at the same dose of atrazine. These findings suggest that the field dose of the fungicide Icarus^®^, containing tebuconazole, may fall within a concentration range that induces LDH activity; on the contrary, the field dose of Mirador^®^, containing azoxystrobin, may fall within a concentration range that inhibits LDH activity.

## 5. Conclusions

In conclusion, this study provided new insights into the sub-lethal effects of two foliar and two ear fungicides, alone and combined, on *E. fetida*, a model organism used for standardized toxicity tests. This work expands the range of biomarkers available for assessing the toxicological effects of agrochemicals on earthworms, covering a broader set of molecular endpoints. No differences among the different treatments for catalase and lipid peroxidation results suggest no oxidative stress after exposure to fungicides. The highest GST activity and DNA fragmentation values found in Amistar^®^Xtra treatment highlight a possible interaction between the active ingredients and unknown co-adjuvants. Similar effects after exposure to the Amistar^®^Xtra-Prosaro^®^ (A-P) mixture were found, confirming the highest toxicity of Amistar^®^Xtra. These results indicate that it is essential to test commercial products and not only the active ingredients, due to the synergistic effects that may amplify their toxicological effects. The present study allows for the hypothesis that exposure to fungicides, both individually and in combination, induces negative effects not only on earthworms but also on other non-target soil organisms, thereby contributing to the degradation of soil ecology within agroecosystems that are already subject to stress conditions arising from intensive management and resource depletion.

## Figures and Tables

**Table 1:** Grain size and physicochemical analysis of the field used for the earthworm’s exposure to the fungicides.

Analysis	Unit of Measure	Value
pH		7.95
Total Nitrogen	N (g/kg)	1.35
Plant-available phosphorus	P_2_O (mg/kg)	14.5
Exchangeable potassium	K_2_O (mg/kg)	147.5
Exchangeable calcium	Ca (mg/kg)	3850.5
Exchangeable magnesium	Mg (mg/kg)	143
Available iron	Fe (mg/kg)	19.5
Organic matter	%	2.28
C/N		9.75
Total lime	%	16.25
Active lime	%	3.5
C.E.C.	meq/100 g	20.8
Clay	%	28
Silt	%	34.4
Sand	%	37.6

## Data Availability

The data are contained within the article. Additional data are available upon request from the corresponding authors.

## Supplementary Materials

The following supporting information can be downloaded at: https://www.mdpi.com/article/10.3390/toxics13030209/s1. Table S1. Results with statistical indices of biomarkers analysis evaluated in E. fetida exposed to foliar fungicides Mirador®, Amistar®Xtra; ear fungicides Icarus®, Prosaro® and the mix Mirador®-Icarus® (M-I), Amistar®Xtra-Prosaro® (A-P).
