# Supplementary material for: Toxicity of Four Commercial Fungicides, Alone and in Combination, on the Earthworm Eisenia fetida: A Field Experiment
Source: Toxics. 2025 Mar 14;13(3):209. doi: 10.3390/toxics13030209 (PMC11945946; doi:10.3390/toxics13030209)
Supplement: Supplementary file 1 [file toxics-13-00209-s001.zip › toxics-3498419-supplementary.pdf]

Toxicity of four commercial fungicides, alone and in combination, on the earthworm *Eisenia fetida*: a field experiment

Tommaso Campani [1], Ilaria Caliani [1*], Agata Di Noi[2] and Silvia Casini[1]

Table S1. Results with statistical indices of biomarkers analysis evaluated in E. fetida exposed to foliar fungicides Mirador®, Amistar®Xtra; ear fungicides Icarus®, Prosaro® and the mix Mirador®-Icarus® (M-I), Amistar®Xtra-Prosaro® (A-P).

**GST** (nmol DNCB x min^{-1} x mg $protein^{-1}$)	**Control**	**Mirador®**	**Amistar®Xtra**	**Icarus®**	**Prosaro®**	**M-I**	**A-P**
N	12	6	8	7	5	7	7
Mean	44.39	51.56	71.62	42.37	38.22	60.57	60.18
Sd	8.80	21.78	14.66	8.14	10.73	19.38	15.03
median	40.86	49.56	71.53	40.83	35.85	57.48	57.00
Min	30.42	28.50	52.02	34.09	29.43	28.99	43.16
Max	59.81	88.65	90.33	55.03	56.25	83.27	85.98
Se	2.54	8.89	5.10	3.08	4.80	7.32	5.68
**CAT** (nmol^{-1} x min)							
N	9	4	7	6	3	6	8
Mean	3.67	4.57	5.03	3.26	4.67	3.72	4.21
Sd	0.92	2.48	2.22	1.02	2.6	1.48	1.79
median	3.65	4.66	4.49	3.42	5.11	4.25	4.09
Min	2.64	1.81	2.94	1.52	1.88	1.26	1.29
Max	5.43	7.17	9.10	4.41	7.02	5.14	7.34
Se	0.31	1.24	0.84	0.41	1.50	0.60	0.63
**LPO**(nmol TBARS x min^{-1} x mg $protein^{-1}$)							
N	13	8	8	8	5	8	8
Mean	1.03	0.97	1.09	1.19	1.33	0.86	1.12
Sd	0.42	0.41	0.67	0.60	0.68	0.41	0.35
median	1.10	0.86	1.27	1.17	1.11	0.80	0.98
Min	0.50	0.53	0.05	0.21	0.51	0.38	0.71
Max	1.58	1.69	1.95	2.12	2.27	1.58	1.74
Se	0.12	0.14	0.24	0.21	0.30	0.14	0.12
**LDH** (μmol^{-1} x min)							
N	3	4	8	7		7	7
Mean	3.98	0.54	2.53	10.91		2.23	14.26
Sd	3.41	0.76	1.98	12.8		2.83	21.77
median	2.58	0.27	2.65	5.11		0.85	5.38
Min	1.48	0.00	0.00	1.62		0.45	0.00
Max	7.86	1.61	4.74	38.35		8.04	59.71
Se	1.97	0.38	0.70	4.84		1.07	8.23
**LYS** (μg/μL)							
N	4	2	6	5		6	5
Mean	2.45	1.91	1.07	3.6		2.32	2.00
Sd	1.86	2.31	0.89	0.91		1.26	1.30
median	2.79	1.91	1.12	3.22		2.25	2.63
Min	0.00	0.27	0.09	2.59		0.79	0.25
Max	4.21	3.54	2.23	4.85		4.18	3.12
Se	0.93	1.63	0.36	0.41		0.51	0.58
**Comet assay** (% DNA tail)							
N	8	6	6	4	4	4	4
Mean	28.60	27.4	36.93	29.11	29.28	31.77	36.87
Sd	3.37	5.93	4.54	3.1	4.66	4.40	3.89

median	28.78	26.9	37.68	30.22	30.02	33.00	37.27
Min	24.22	18.51	31.22	31.22	23.58	25.49	32.46
Max	32.41	34.26	43.32	43.32	33.49	35.6	40.49
Se	1.19	2.42	1.85	1.85	2.33	2.20	1.95
